# Independent Components of EEG Activity Correlating with Emotional State

**DOI:** 10.3390/brainsci10100669

**Published:** 2020-09-25

**Authors:** Yasuhisa Maruyama, Yousuke Ogata, Laura A. Martínez-Tejada, Yasuharu Koike, Natsue Yoshimura

**Affiliations:** 1 Institute of Innovative Research, Tokyo Institute of Technology, Yokohama, Kanagawa 226-8503, Japan; maruyama.y.ae@m.titech.ac.jp (Y.M.); y.ogata1110@gmail.com (Y.O.); martinez.l.aa@m.titech.ac.jp (L.A.M.-T.); koike@pi.titech.ac.jp (Y.K.); 2 Department of Advanced Neuroimaging, Integrative Brain Imaging Center, National Center of Neurology and Psychiatry, Kodaira, Tokyo 187-8551, Japan; 3 PRESTO, JST, Kawaguchi, Saitama 332-0012, Japan; 4 Neural Information Analysis Laboratories, ATR, Kyoto 619-0288, Japan

**Keywords:** brain-computer interface (BCI), electroencephalography (EEG), emotion recognition, independent component analysis (ICA), regression

## Abstract

Among brain-computer interface studies, electroencephalography (EEG)-based emotion recognition is receiving attention and some studies have performed regression analyses to recognize small-scale emotional changes; however, effective brain regions in emotion regression analyses have not been identified yet. Accordingly, this study sought to identify neural activities correlating with emotional states in the source space. We employed independent component analysis, followed by a source localization method, to obtain distinct neural activities from EEG signals. After the identification of seven independent component (IC) clusters in a k-means clustering analysis, group-level regression analyses using frequency band power of the ICs were performed based on Russell’s valence–arousal model. As a result, in the regression of the valence level, an IC cluster located in the cuneus predicted both high- and low-valence states and two other IC clusters located in the left precentral gyrus and the precuneus predicted the low-valence state. In the regression of the arousal level, the IC cluster located in the cuneus predicted both high- and low-arousal states and two posterior IC clusters located in the cingulate gyrus and the precuneus predicted the high-arousal state. In this proof-of-concept study, we revealed neural activities correlating with specific emotional states across participants, despite individual differences in emotional processing.

## 1. Introduction

**Figure 1:**
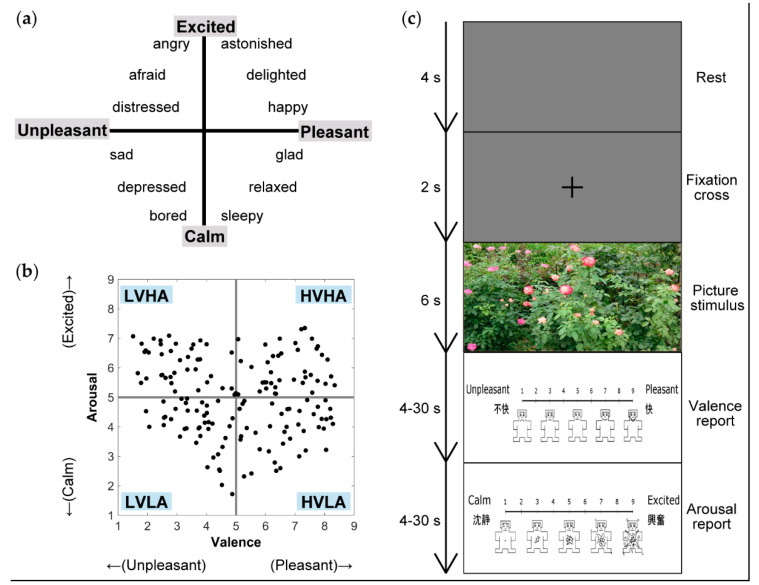
Experimental settings. (**a**) Schema of Russell’s valence–arousal model [5]. (**b**) The distribution of the International Affective Picture System normative ratings of the 160 pictures used in the current study. On the valence axis, values of 9, 5, and 1 represent pleasant, neutral, and unpleasant states, respectively. On the arousal axis, values of 9, 5, and 1 represent excited, neutral, and calm states, respectively. HVHA, high valence and high arousal; HVLA, high valence and low arousal; LVHA, low valence and high arousal; LVLA, low valence and low arousal. (**c**) Trial flow. Each trial consisted of 4 s of rest, 2 s of fixation cross presentation, 6 s of picture stimulus presentation, and 4–30 s of reporting of the felt valence and arousal levels. Valence and arousal levels were reported using a computerized visual analog scale with a self-assessment manikin (SAM; [11]). In the valence and arousal reporting scales, the emotions were written in English and Japanese at both ends of the scales.

Emotion plays an important role in daily life, because it enriches communication. To achieve emotional interaction between human beings and computers, electroencephalography (EEG)-based emotion recognition is gaining attention in brain-computer interface (BCI) studies. For engineering purposes, many studies have performed emotion classification using various types of EEG features [1,2,3]. In [4], emotion classification based on Russell’s valence–arousal model was performed using event-related potentials (ERPs) and event-related oscillations calculated from EEG recorded during affective picture viewing. Russell’s valence–arousal model is a widely-recognized model of emotion, and in this model, emotions are represented in the space of two axes: valence (ranging from pleasant to unpleasant state) and arousal (ranging from excited to calm state), as illustrated in Figure 1a [5]. Among many emotion classification studies based on EEG, some studies estimated source activities in the brain and performed classification using only emotion-related source signals [6,7,8,9]. Padilla-Buritica and colleagues reconstructed source-level signals from scalp EEG and classified emotions using signals extracted from the selected brain regions [6]. Their results showed improvement of prediction accuracy by estimation of source signals combined with the appropriate selection of brain regions from which features used in the classification analysis were computed. Additionally, in EEG-based emotion recognition studies, identification of common EEG features across individuals is required to create models that can be generalized to various people, although there are large individual differences in emotional processing and many studies tested participant-dependent models [10].

In addition to emotion classification studies which are based on a limited number of pre-defined emotional categories, several studies have performed EEG-based or electrocorticography (ECoG)-based emotion or mood state regression/correlation analyses to recognize small-scale emotional changes [12,13,14,15,16,17,18,19,20,21,22,23,24,25,26,27,28,29,30]. McFarland and colleagues performed canonical correlation analysis (CCA) to predict participants’ emotional states from the sensor-level features of EEG recorded during affective picture viewing [22]. Their results showed difficulty in attaining good prediction accuracy in the test set. Similar to the classification analyses, the use of source-level signals might improve the prediction accuracy in regression analysis. However, to the best of our knowledge, no study has identified effective brain regions in EEG-based emotion regression.

To estimate source-level neural activities, independent component analysis (ICA) with subsequent dipole fitting has often been used. ICA is a signal separation method that linearly decomposes multi-channel data into independent signals [31]. The application of ICA to EEG data enables extraction of distinct neural activities that are free from any type of noise and whose activities are independent of each other. Localization of the sources of independent components (ICs) can be performed based on their projections onto the scalp, which allows investigation at the source level. ICA with subsequent source estimation methods has often been applied in EEG studies investigating emotional processing in the brain [32,33,34,35,36,37,38], suggesting the suitability of this method for the identification of effective brain regions in EEG-based emotion regression.

Accordingly, this proof-of-concept study elucidated brain regions that recognize small-scale emotional changes by using regression analysis. We applied ICA to EEG data captured during affective picture viewing and then grouped the obtained ICs into IC clusters by a k-means clustering method. In the regression analyses, valence and arousal levels were predicted from theta, alpha, beta, and gamma band power of ICs, since frequency band power has been one of the most popular features in EEG-based emotion classification studies [2]. A leave-one-participant-out approach was used to identify effective features that are common across participants, and emotional state-specific regression analyses were introduced with the aim of improving regression performance based on functional magnetic resonance imaging (fMRI) studies suggesting difference of the neural activities between high- and low-valence states and between high- and low-arousal states [39,40].

## 2. Materials and Methods

### 2.1. Participants

Twenty-six healthy human participants (10 females; mean age [standard deviation (S.D.)]: 25.2 [4.1] years) with normal or corrected-to-normal vision participated in this study. All participants were right-handed and had no history of psychiatric or neurological disorders. Data obtained from 1 male participant were excluded from the analysis because of an unacceptably noisy signal. The experimental protocol was approved by the ethics committee of the Tokyo Institute of Technology (Approval No. A17011) and conducted in accordance with the Declaration of Helsinki. The procedures were explained to each participant and written informed consent was obtained prior to the experiment.

### 2.2. Stimuli

Pictures selected from the International Affective Picture System (IAPS; [41]) were used as stimuli to induce emotion. The IAPS is commonly used in research on emotion. It provides general information on the emotion induced by each picture stimulus, since all IAPS pictures have normative ratings of valence, arousal, and dominance (ranging from controlled to in-control) levels. These ratings are mean values of multiple participants’ ratings obtained in previous research. Figure 1b illustrates the distribution of normative ratings of IAPS pictures used in this study in the valence–arousal plane. Both valence and arousal axes ranged from 1 to 9 (9 = pleasant in the valence axis and excited in the arousal axis, 1 = unpleasant in the valence axis and calm in the arousal axis). A value of 5 represented a neutral level in both axes. For this study, pictures covering a wide area of the valence–arousal plane were selected. Hereafter, we refer to values higher or lower than 5 in valence or arousal ratings as belonging to a high or low emotional state, respectively. We selected 160 pictures distributed equally in each of the four quadrants (40 pictures per quadrant: high valence and high arousal [HVHA], high valence and low arousal [HVLA], low valence and high arousal [LVHA], and low valence and low arousal [LVLA]).

### 2.3. Experimental Task

Participants were positioned sitting in a reclining chair in a sound-attenuated chamber and instructed to look at a monitor positioned approximately 1 m away from their eyes during the experiment. Each trial consisted of 4 s of rest, 2 s of fixation cross presentation, 6 s of picture stimulus presentation, and 2 reporting periods (Figure 1c). During the reporting periods, participants were asked to report the valence and arousal levels they felt during the presentation of the picture stimulus by using a computerized visual analog scale ranging from 1 to 9 (in 0.1-point steps) and a touch pad, within 30 s. The minimum reporting time was set to 4 s to ensure that the participants were reporting accurately, and not perfunctorily. A self-assessment manikin (SAM; [11]) was placed below the visual analog scales to assist the participants in reporting. We instructed the participants not to think about the reporting periods during picture stimulus presentation in order to avoid such thoughts influencing EEG signals during picture stimulus viewing. The experiment consisted of eight sessions, with each session comprising 20 trials. The picture stimuli were presented in a pseudo-random order, so that each of the 4 quadrants (HVHA, HVLA, LVHA, and LVLA) consisted of 5 pictures presented in 1 session, thereby, equalizing the emotions induced by the stimuli across the sessions. Participants were allowed to take breaks of unlimited duration between sessions. Before the experiment, we explained the meanings of the valence and arousal axes to the participants and allowed them to rehearse using three practice picture stimuli that were not used in the subsequent experiment. All images were presented using a 24-inch monitor connected to a computer, and MATLAB R2018b (The MathWorks, Inc., Natick, MA, USA) and Psychophysics toolbox [42,43,44] were used to control the experimental program.

### 2.4. EEG Data Acquisition

EEG signals were recorded from 64 channels using a Biosemi Active Two amplifier system with active sensors (Biosemi, Amsterdam, Netherlands) at a sampling rate of 2048 Hz. All EEG channels were attached to the participant’s scalp using electrically conductive gel, according to the International 10–20 system, with two reference channels attached to their earlobes.

### 2.5. EEG Data Processing

**Figure 2:**
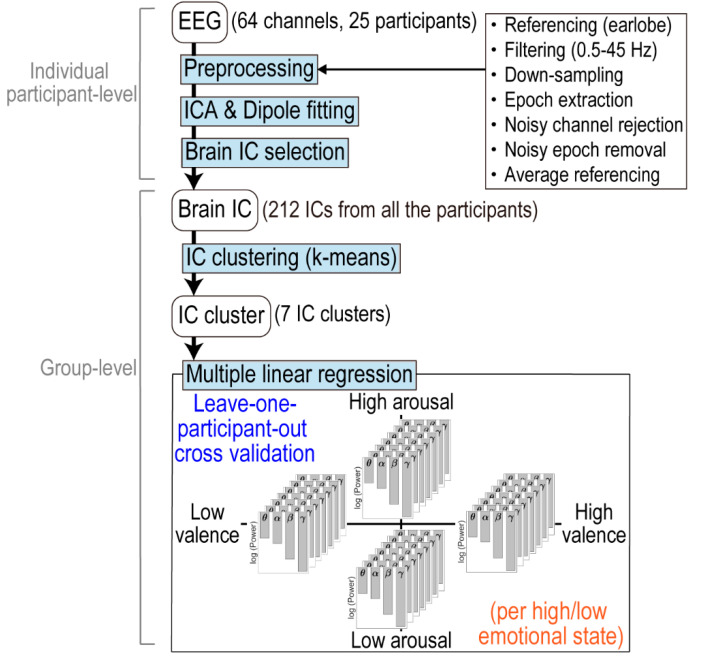
Flow of data processing and analysis. In the regression analysis, valence and arousal levels were predicted by inter-participant regression from logarithms of theta (4–7 Hz), alpha (8–13 Hz), beta (14–30 Hz), and gamma (31–45 Hz) band power of independent components (ICs).

EEG data were processed using MATLAB R2018b and EEGLAB 14.1.2 software [45] (Figure 2). First, we set the reference signal as the average of the signals at both earlobes. We applied a high-pass finite impulse response (FIR) filter at 0.5 Hz and a low-pass FIR filter at 45 Hz to the raw continuous 64-channel data to attenuate the noise, and subsequently down-sampled the signal to 512 Hz to reduce the computational cost. To clean the data, we rejected and interpolated noisy channels based on visual inspection after extracting the 6-s epochs of picture stimulus presentation. Then, we removed epochs containing artefacts, such as muscle activities, by visual inspection, and concatenated all the remaining epochs. On average, 1.04 channels were rejected and 5.2% of epochs were removed. Following this preprocessing, we applied adaptive mixture ICA (AMICA, https://sccn.ucsd.edu/~jason/amica_web.html; [46]) to the data after changing the reference to the average of all 64-channel signals and reducing the data dimensions to their rank by principal component analysis. Subsequently, we used the DIPFIT3 function (https://sccn.ucsd.edu/wiki/A08:_DIPFIT) of the FieldTrip toolbox [47] to locate the equivalent current source dipole of each IC, based on boundary element model (BEM) of the Montreal Neurological Institute (MNI) standard brain. To perform the analysis using only ICs originating from brain activity, we identified and extracted the brain ICs. In this evaluation, we excluded ICs whose residual variances in the dipole fitting procedure exceeded 15% [31], those with dipole locations that were localized outside the brain, or those whose characteristics such as power spectral densities (PSDs) and time series activities across epochs (ERP images) did not appear to be brain activities by visual inspection. In total, 212 ICs (between 3 and 14 ICs per participant, mean 8.48) were selected as brain ICs.

Finally, we conducted a cluster analysis to identify common ICs across participants by using a k-means clustering method in EEGLAB. All brain ICs were clustered by their characteristics, such as scalp topographies, dipole locations, and ERPs in 0–500 milliseconds. To reduce the total number of the dimension, principal component analysis was performed on each of the scalp topography and ERP data, and top 10 and 5 principal components were included in the cluster analysis, respectively. Accordingly, the k-means clustering analysis was performed in the 18-dimensional space (3 dimensions for the dipole locations, 10 for the scalp topography, and 5 for the ERP). The number of clusters (k) was set to a value that we considered to yield plausible clustering results in terms of consistency of characteristics across ICs within the same cluster and distinctness of characteristics between different IC clusters, after several changes in k. The threshold level for outliers was set to 3 S.D., and only IC clusters with ICs present in more than half of all participants (i.e., 13) were reported. IC clusters were labeled using automated anatomical labeling (AAL; [48]) in MRIcron software (http://people.cas.sc.edu/rorden/mricron/index.html; [49]), based on the MNI coordinates of centroids of IC clusters. If the coordinates were judged to be in the white matter by AAL, the nearest labels in the MNI space were reported.

### 2.6. Regression Analyses

The regression analysis was performed in a leave-one-participant-out manner and per emotional state: high-valence, low-valence, high-arousal, or low-arousal state. We predicted the valence and arousal levels by multiple linear regression with the ordinary least-squares method from logarithms of theta (4–7 Hz), alpha (8–13 Hz), beta (14–30 Hz), and gamma (31–45 Hz) band power of ICs included in the IC clusters. PSDs of 6-s epochs of each IC were calculated by 512-point (1-s) fast Fourier transform (FFT) with a 128-point (0.25-s) overlap, and the Hann window was applied as the window function. When more than 2 ICs from 1 participant were included in an IC cluster, their PSDs were averaged before logarithmic transformation.

Based on fMRI studies suggesting that some brain regions are differently activated in high- and low-valence (pleasant and unpleasant) states or in high- and low-arousal (excited and calm) states during affective picture processing [39,40], we performed four separate regression analyses for each IC cluster. Specifically, (1) regression of the valence level in a high-valence state (using HVHA and HVLA pictures in Figure 1b, (2) regression of the valence level in a low-valence state (using LVHA and LVLA pictures), (3) regression of the arousal level in a high-arousal state (using HVHA and LVHA pictures), and (4) regression of the arousal level in a low-arousal state (using HVLA and LVLA pictures) were performed. For dependent variables, we used the IAPS normative ratings of each picture stimulus rather than the participants’ reports, because some participants’ reports were biased to either high- or low- valence/arousal state. In these participants, only a few pictures were included in the other emotional state, based on the participants’ reports. In order to ensure adequate sample sizes for the regression analyses and to equalize sample sizes across participants and across emotional states, we determined to use IAPS normative ratings as the dependent variable. The prediction accuracy was evaluated using Pearson’s correlation coefficient between predicted variables and IAPS normative ratings. We conducted a leave-one-participant-out cross validation and reported the mean values of Pearson’s correlation coefficients obtained with all participants whose ICs were included in the IC cluster. Before regression analyses, all independent and dependent variables for each participant were standardized to mean values of 0 and variances of 1.

### 2.7. Identification of IC Clusters Correlating with Emotional State

We identified IC clusters that exhibited Pearson’s correlation coefficients significantly higher than 0 in each emotional state for each dependent variable (valence or arousal level) using a one-tailed one-sample *t*-test. For each combination of the dependent variable and the emotional state, the obtained *p*-values were corrected with the Holm–Bonferroni method to compensate for multiple comparisons across the seven identified IC clusters [50]. As identification of significant IC clusters was performed in two emotional states per dependent variable, the significance level was set to 0.025 (=0.05/2). Furthermore, we calculated the coefficients of the regression model in which successful prediction was obtained to determine the degree of contribution of each frequency band. Statistical tests and calculation of mean correlation coefficients were performed after transformation to Fisher’s z values.

## 3. Results

### 3.1. IC Clusters Obtained by ICA and Cluster Analysis

**Figure 3:**
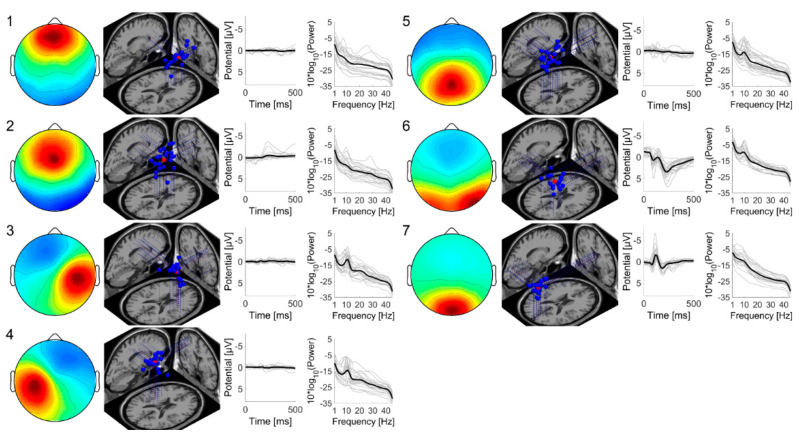
Average projections onto the scalp, dipole locations in the Montreal Neurological Institute (MNI) brain (blue points: each dipole, red points: centroid of dipoles), event-related potentials (ERPs) during 0–500 milliseconds, and power spectral densities (PSDs) in 1–45 Hz of each IC cluster obtained from independent component analysis and cluster analysis by a k-means clustering method in EEGLAB. The numbers from 1 to 7 represent cluster indices shown in Table 1. In the scalp topography maps, the left and right sides represent left and right hemispheres, respectively. The upper and lower sides represent anterior and posterior sides of the scalp, respectively. The red color represents positive weight and the blue color represents negative weight. In plots of ERPs and PSDs, thin gray lines represent data from each IC and thick black lines represent averaged values.

ICA and cluster analysis by a k-means clustering method resulted in seven IC clusters. The MNI coordinates of their centroids and their labels are presented in Table 1. The average projections of the ICs onto the scalp, their dipole locations in the MNI standard brain, ERPs during 0–500 milliseconds, and PSDs in 1–45 Hz are illustrated in Figure 3. Two anterior IC clusters (IC clusters 1 and 2) were located in the anterior cingulate gyrus and middle cingulate gyrus. Two additional lateral IC clusters (IC clusters 3 and 4) were located in the right and left precentral gyrus. Both of these lateral IC clusters showed prominent alpha peaks in their PSDs. The other 3 IC clusters, clusters 5, 6, and 7, were located in the posterior part of the brain, middle cingulate gyrus, right precuneus, and cuneus, respectively. IC clusters 6 and 7 had specific ERPs, and IC clusters 5 and 6 exhibited alpha peaks in their PSDs.

### 3.2. IC Clusters Correlating with Emotional State

**Figure 4:**
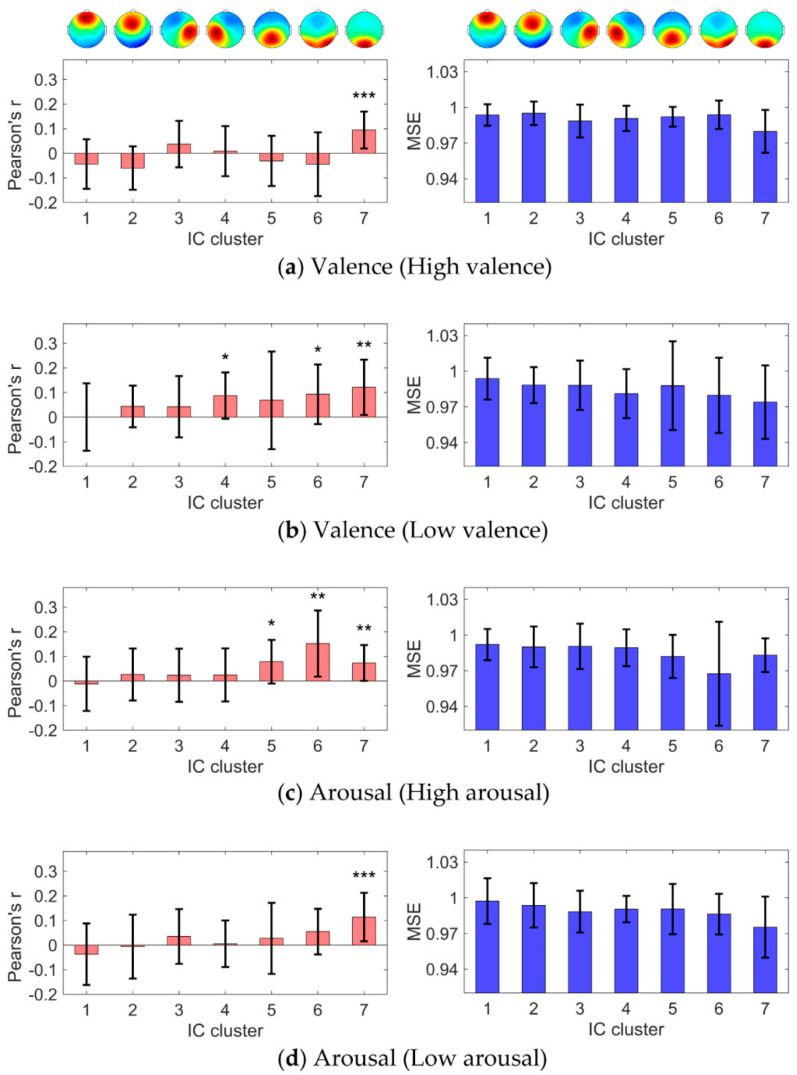
Prediction accuracy of the seven identified IC clusters in the inter-participant regression. Error bars represent standard deviations. (**a**) Regression of the high-valence state. (**b**) Regression of the low-valence state. (**c**) Regression of the high-arousal state. (**d**) Regression of the low-arousal state. (Left column) Pearson’s correlation coefficient between predicted values and International Affective Picture System normative ratings. (Right column) mean squared error (MSE). * *p* < 0.025, ** *p* < 0.005, *** *p* < 0.0005 in one-tailed one-sample t-tests with Holm–Bonferroni correction.

Prediction accuracy (Pearson’s correlation coefficient between predicted values and IAPS normative ratings and mean squared error (MSE)) of all IC clusters in the inter-participant regression analysis is shown in Figure 4. Among the 7 IC clusters, 1, 3, 3, and 1 IC clusters reached significance in the regression of the high-valence, low-valence, high-arousal, and low-arousal states, respectively.

**Figure 5:**
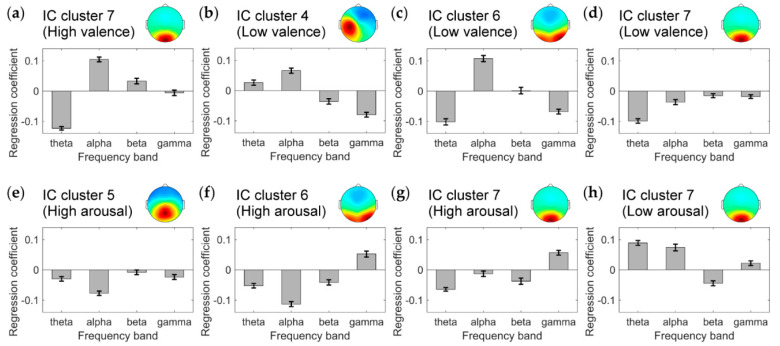
Mean coefficients of the theta, alpha, beta, and gamma bands in the regression model. Larger absolute value of the regression coefficient indicates larger degree of contribution of the frequency band to the regression model. Positive (negative) regression coefficient indicates positive (negative) relationship between the frequency band power and IAPS normative ratings. Error bars represent standard deviations. (**a**) Regression of the high-valence state of IC cluster 7. (**b**) Low-valence state of IC cluster 4. (**c**) Low-valence state of IC cluster 6. (**d**) Low-valence state of IC cluster 7. (**e**) High-arousal state of IC cluster 5. (**f**) High-arousal state of IC cluster 6. (**g**) High-arousal state of IC cluster 7. (**h**) Low-arousal state of IC cluster 7.

In the regression analysis of the high-valence state, IC cluster 7 (with the centroid located in the cuneus) exhibited a correlation coefficient significantly higher than 0 (mean correlation coefficient: 0.095, *p* < 0.0005 in a one-tailed t-test with Holm–Bonferroni correction; MSE: 0.980). Mean (S.D.) regression coefficients of the theta, alpha, beta, and gamma bands were −0.12 (0.01), 0.10 (0.01), 0.03 (0.01), and −0.01 (0.01), respectively (Figure 5a).

In the regression analysis of the low-valence state, IC clusters 4 (with the centroid located in the left precentral gyrus), 6 (with the centroid located in the precuneus), and 7 exhibited correlation coefficients significantly higher than 0. Mean correlation coefficients were 0.088 (*p* < 0.025), 0.093 (*p* < 0.025), and 0.121 (*p* < 0.005), respectively, and MSEs were 0.981, 0.980, and 0.974, respectively. The mean (S.D.) regression coefficients of the theta, alpha, beta, and gamma bands were 0.03 (0.01), 0.07 (0.01), −0.04 (0.01), and −0.08 (0.01), respectively, in IC cluster 4 (Figure 5b), −0.10 (0.01), 0.11 (0.01), 0.00 (0.01), and −0.07 (0.01), respectively, in IC cluster 6 (Figure 5c), and −0.10 (0.01), −0.04 (0.01), −0.02 (0.01), and −0.02 (0.01), respectively, in IC cluster 7 (Figure 5d).

In the regression analysis of the high-arousal state, IC clusters 5 (with the centroid located in the middle cingulate gyrus), 6, and 7 exhibited correlation coefficients significantly higher than 0. Mean correlation coefficients were 0.079 (*p* < 0.025), 0.152 (*p* < 0.005), and 0.074 (*p* < 0.005), respectively, and MSEs were 0.982, 0.967, and 0.983, respectively. The mean (S.D.) regression coefficients of the theta, alpha, beta, and gamma bands were −0.03 (0.01), −0.08 (0.01), −0.01 (0.01), and −0.02 (0.01), respectively, in IC cluster 5 (Figure 5e), −0.05 (0.01), −0.11 (0.01), −0.04 (0.01), and 0.05 (0.01), respectively, in IC cluster 6 (Figure 5f), and −0.06 (0.01), −0.01 (0.01), −0.04 (0.01), and 0.06 (0.01), respectively, in IC cluster 7 (Figure 5g).

In the regression of the low-arousal state, IC cluster 7 exhibited a correlation coefficient significantly higher than 0 (mean correlation coefficient: 0.114, *p* < 0.0005; MSE: 0.975). Mean (S.D.) regression coefficients of the theta, alpha, beta, and gamma bands were 0.09 (0.01), 0.07 (0.01), −0.04 (0.01), and 0.02 (0.01), respectively (Figure 5h). The other 3 IC clusters (IC clusters 1, 2, and 3) did not exhibit correlation coefficients significantly higher than 0 for either dependent variable (valence or arousal level).

## 4. Discussion

In this study, we investigated neural activities that can be recorded from scalp EEG and correlate with emotional states, by using ICA with dipole fitting and regression analysis. We first identified seven IC clusters in the frontal, parietal, and occipital regions in the group analysis. These clusters were distinct from each other in terms of scalp topography, MNI coordinates, and ERPs. Subsequently, inter-participant emotion regression was performed using frequency band power of the ICs included in the seven IC clusters. As a result, the relationship between specific emotional states and four IC clusters was identified in spite of individual differences in emotional processing [51]. In the regression of the valence level, we found that an IC cluster located in the cuneus (IC cluster 7) predicted both high- and low-valence states and two other IC clusters located in the left precentral gyrus and the precuneus (IC clusters 4 and 6) predicted the low-valence state. In the regression of the arousal level, IC cluster 7 was found to also predict both high- and low-arousal states and 2 posterior IC clusters located in the cingulate gyrus and the precuneus (IC clusters 5 and 6) were found to predict the high-arousal state. Thus, the results suggest that these brain regions are good candidates for effective brain regions in EEG-based emotion regression.

In the regression of the valence level, IC cluster 7 (located in the cuneus) showed significant regression performance in both high- and low-valence states. The cuneus is located in the occipital lobe and is thought to be strongly related to visual function. This IC cluster also exhibited specific ERPs, comprising P1, N1, and P2 in the 100–250 milliseconds range. Based on the ERP and the location, this IC cluster may reflect the activity related to early visual processing. The activity of the visual cortex has been found to be affected by emotion [52]. The regression coefficients of the frequency bands suggest a strong influence of the theta band power. In human EEG studies, increased theta band response to affective picture stimuli than to neutral ones has been reported at the posterior electrodes [53,54], and these theta band modulations were suggested to reflect top-down and bottom-up attentional mechanisms [55,56]. Attention-mediated theta band power change in the visual cortex was also shown in a macaque ECoG study [57]. Thus, the regression model in our study might decode attention-related neural activity. However, our results showed that the direction of the theta band-power influence differed between high- and low-valence states. While theta band power decreased as the pleasantness increased in the high-valence state, theta band power increased as the unpleasantness increased in the low-valence state. (In the valence axis, a value of 1 represents an unpleasant state, and a value of 5 represents a neutral state in the regression model; thus, the negative coefficient indicates a positive correlation with unpleasantness in the low-valence state.) This may indicate different mechanisms of emotional processing in the cuneus between these two emotional states. In addition, IC cluster 6 (located in the right precuneus) significantly contributed to the regression, but only for the low-valence state. The precuneus is located in the parietal lobe and is thought to be involved in visuo-spatial imagery, episodic memory retrieval, and self-processing [58]. In our study, IC cluster 6 had specific ERPs, comprising P300 and the subsequent late positive potential (LPP). Therefore, this IC cluster may represent neural activation related to attention and memory processing, since P300 and LPP are thought to be associated with these processes [59,60,61,62]. In particular, a larger LPP amplitude is thought to be associated with increased motivated attention and memory. In a study using simultaneous EEG and fMRI measurements, Liu and colleagues demonstrated that the amplitude of LPP significantly positively correlated with blood-oxygenation-level-dependent (BOLD) signals in the precuneus only in the presentation of high-arousal (“pleasant” and “unpleasant” in their study) pictures [63]. However, we observed significant results in regression analysis of the low-valence state, but not of the high-valence state. This result may be partly due to the “negativity bias”; it has been suggested that unpleasant stimuli induce stronger emotional responses in the brain than pleasant stimuli [64]. Accordingly, although both pleasant and unpleasant stimuli induced emotional responses in the brain, the unpleasant stimuli might induce attention and memory processing more strongly; thus, the activity could be strongly represented in the EEG. In addition, Liu et al. found that the LPP correlated with the BOLD signal in the ventral part of the precuneus in the “unpleasant” condition and in the more dorsal part of the precuneus in the “pleasant” condition. The location of IC cluster 6 in our study is in the ventral part of the precuneus, near the posterior cingulate cortex. Taken together, activity in the ventral part of the precuneus may correlate with the low-valence state. Moreover, IC cluster 4 (located in the left precentral gyrus) also significantly contributed to the regression of the low-valence state. The precentral gyrus is mainly associated with motor function; thus, this IC cluster may be related to motor activity caused by the emotional content of the picture stimuli. One type of the motor activity may be induced through facial muscles, because negative emotions, such as fear, anger, sadness, and disgust, are accompanied by unique facial expressions [65]. Involvement of the precentral gyrus in the low-valence state is in accordance with an fMRI study using affective pictures [39]. The regression coefficients of the frequency bands support the contribution of motor activity to the regression performance. In the regression model, the alpha and gamma bands contributed most significantly; as unpleasantness increased, alpha band power decreased and gamma band power increased. Movement accompanies a decrease in alpha band power in the central area, in a process called event-related desynchronization [66]. A previous human ECoG study has reported the relationship between gamma band power and movement execution [67]. Accordingly, movement-related activity of IC cluster 4 might correlate with the level of unpleasantness.

In the regression of the arousal level, IC cluster 5 (located in the middle cingulate gyrus; Brodmann area [BA] 31) significantly predicted the high-arousal state. This IC cluster was located in the posterior part of the brain, and the posterior cingulate cortex, including BA31, has been associated with controlling attentional focus [68]. IC cluster 6 located in the right precuneus also significantly contributed to the regression, but only for the high-arousal states. As mentioned above, a study using simultaneous EEG and fMRI measurements demonstrated the contribution of the precuneus to the high-arousal state [63]. In accordance with the results of the current study, they observed no such correlation in the presentation of low-arousal (“neutral” in their study) pictures. Their results suggest that the degree of activation in the precuneus during affective picture viewing correlates with the electrophysiological index of motivated attention and memory, but only in the high-arousal state. The regression coefficients of the frequency bands also supported a contribution of visual attention in IC clusters 5 and 6 to the regression performance. In the regression analysis of the high-arousal state in IC clusters 5 and 6, the alpha band power contributed most significantly. The result suggested that, as arousal level increased, alpha band power decreased. It has been suggested that alpha band power in the parietal–occipital region is associated with visual attention [69]. Taken together, these findings suggest that attention and memory processing are key factors that correlate with the high-arousal states in these two posterior IC clusters. Additionally, IC cluster 7 showed significant regression performance in both high- and low-arousal states. However, as with the valence axis, mechanisms of emotional processing may be different between high- and low-arousal states in the cuneus. Specifically, theta band power decreased as the arousal level increased in the high-arousal state and theta band power increased as the arousal level increased in the low-arousal state.

Among the 3 posterior IC clusters (IC clusters 5, 6, and 7), IC cluster 7 exhibited activity correlating with both high- and low-valence and high- and low-arousal states, while IC clusters 5 and 6 predicted only either half of the valence and/or arousal axes. This may indicate that neural activity responsible for early visual processing correlated with valence and arousal levels regardless of emotional states, while those that process more higher-level information showed emotional state-specific activities. Based on our current results suggesting the existence of neural activities involved in emotion regression only in specific emotional states and fMRI studies showing different neural activities between high- and low-valence states and between high- and low-arousal states [39,40], applying separate regression models to high- and low- valence/arousal states would result in higher prediction accuracy in EEG-based emotion regression analysis. Additionally, although posterior IC clusters correlated with emotional states, 2 anterior IC clusters (IC clusters 1 and 2) did not predict either valence or arousal level. This may be because frontal regions are responsible for more complicated functions than simple visual processing. Frontal regions are thought to have a general role in emotional processing [70] and to be a place of higher cognitive function [71]. Accordingly, in contrast to the posterior IC clusters that seem to be responsible for rather simple functions, anterior IC clusters did not exhibit correlation with emotional states.

Though we identified neural activities correlating with specific emotional states by applying separate regression models per emotional state, there are some potential limitations in this study. The first limitation is the low prediction accuracy. The correlation coefficients were 0.15 at the most in this study. However, this value is higher than that of the previous study performed by McFarland and colleagues, where sensor-level features and whole-axis analyses were used and the correlation coefficients were at most around 0.08 in the test data [22]. Since experimental and analytical procedures were totally different in many respects, it is not possible to directly compare our results with those of the previous study. Nevertheless, our results may suggest the effectiveness of the use of source-level signals in EEG-based emotion regression and/or the existence of neural activities correlating with only specific emotional states. These findings would be useful for establishing regression models that can achieve high prediction accuracy in future research. Moreover, to further increase the prediction accuracy for the real-life application of EEG-based emotion regression, non-linear regression methods may be effective. Specifically, deep neural networks would result in significant improvement of the regression performance [30]. Additionally, use of connectivity and causality measures between brain regions rather than focusing on single brain region would also be beneficial. The second limitation is the dependent variables used in this study. We did not use the participants’ reports, but rather utilized IAPS normative ratings to maintain the large sample sizes and to equalize the sample sizes across participants and across emotional states. Though IAPS normative ratings highly correlated with the participants’ reports in this study (mean Spearman’s rank correlation coefficients across the participants were 0.72 in the valence axis and 0.53 in the arousal axis), IAPS normative ratings may differ from the participants’ actual emotion to some extent. Thus, regression performance would be higher if we could use a larger sample and utilize the participants’ reports as the dependent variable, though Petrantonakis and Hadjileontiadis reported that using participants’ reports instead of IAPS normative ratings did not increase the prediction accuracy in emotion classification [72,73]. The third limitation is the possible effects of visual features of the affective pictures on the regression performance. We found that occipital IC cluster (IC cluster 7) predicted the emotional states; however, the visual cortex processes various visual features. Though the visual cortex has been reported to be involved in emotional processing [52], this IC cluster might respond to physical properties of the pictures rather than the emotional content.

## 5. Conclusions

In order to identify neural activities that can predict valence and arousal levels, we used ICA followed by the source localization method and inter-participant regression analyses. As a result, 4 IC clusters correlated with certain emotional states in spite of individual differences in emotional processing. The results were physiologically plausible and in line with those of previous studies. Specifically, attention and memory processing might contribute to the significant regression results in three posterior IC clusters. Finally, this study suggested that applying separate regression models based on the target emotional states would help to attain good prediction accuracy in EEG-based emotion regression research.

## Figures and Tables

**Table 1:** Montreal Neurological Institute (MNI) coordinates and labels of the centroids of independent component (IC) clusters.

Cluster Index	Location of Centroid ^1^	MNI Coordinates (X, Y, Z)	Number of Participants	Number of ICs
1	Right anterior cingulate gyrus	(2, 39, −2)	14	21
2	Right middle cingulate gyrus	(1, −5, 32)	14	21
3	Right precentral gyrus	(39, −6, 36)	16	18
4	Left precentral gyrus	(−28, −13, 43)	16	19
5	Left middle cingulate gyrus	(−3, −40, 44)	16	24
6	Right precuneus	(20, −45, 4)	18	19
7	Right cuneus	(6, −84, 16)	19	19

^1^ Labels were determined using automated anatomical labeling (AAL; [48]).
